# *IL7RA* rs6897932 Polymorphism Is Associated with Better CD4^+^ T-Cell Recovery in HIV Infected Patients Starting Combination Antiretroviral Therapy

**DOI:** 10.3390/biom9060233

**Published:** 2019-06-16

**Authors:** Salvador Resino, María A. Navarrete-Muñoz, Julià Blanco, Yolanda M. Pacheco, Iván Castro, Juan Berenguer, Jesús Santos, Francisco J. Vera-Méndez, Miguel Górgolas, M. A. Ángeles Jiménez-Sousa, José M. Benito, Norma Rallón

**Affiliations:** 1 Unidad de Infección Viral e Inmunidad, Centro Nacional de Microbiología, Instituto de Salud Carlos III, 28220 Majadahonda, Spain; majimenezsousa@yahoo.es; 2 HIV and Viral Hepatitis Research Laboratory, Instituto de Investigación Sanitaria Fundación Jiménez Díaz, Universidad Autónoma de Madrid (IIS-FJD, UAM), 28040 Madrid, Spain; maria.navarretemu@hospitalreyjuancarlos.es (M.A.N.-M.); normaibon@yahoo.com (N.R.); 3 Hospital Universitario Rey Juan Carlos, 28933 Móstoles, Spain; 4 IrsiCaixa AIDS Research Institute, 08916 Badalona, Spain; jblanco@irsicaixa.es; 5 Laboratory of Immunology, Instituto de Biomedicina de Sevilla (IBiS)/UGC Clinical Laboratories, Hospital Universitario Virgen del Rocío, 41013 Sevilla, Spain; ypacheco-ibis@us.es; 6 Hospital Universitario y Politécnico de La Fe, 46026 Valencia, Spain; icasher86@gmail.com; 7 Hospital General Universitario Gregorio Marañón, 28007 Madrid, Spain; jbb4@me.com; 8 Hospital Universitario Virgen de la Victoria, 29010 Málaga, Spain; med000854@gmail.com; 9 Instituto de Investigación Biomédica de Málaga (IBIMA), 29010 Málaga, Spain; 10 Hospital Universitario Santa Lucía, 30202 Cartagena, Spain; franciscovera72@gmail.com; 11 Hospital Universitario Fundación Jiménez Díaz, 28040 Madrid, Spain; MGorgolas@fjd.es

**Keywords:** HIV, IL7RA, SNPs, immune reconstitution, CD4, cART

## Abstract

Interleukin-7 receptor subunit alpha (*IL7RA*) rs6897932 polymorphism IS related to CD4^+^ recovery after combination antiretroviral therapy (cART), but no studies so far have analyzed its potential impact in patients with very low CD4^+^ T-cells count. We aimed to analyze the association between *IL7RA* rs6897932 polymorphism and CD4^+^ T-cells count restoration in HIV-infected patients starting combination antiretroviral therapy (cART) with CD4^+^ T-cells count <200 cells/mm^3^. We performed a retrospective study in 411 patients followed for 24 months with a DNA sample available for genotyping. The change in CD4^+^ T-cells count during the follow-up was considered as the primary outcome. The rs6897932 polymorphism had a minimum allele frequency (MAF) >20% and was in Hardy–Weinberg equilibrium (*p* = 0.550). Of 411 patients, 256 carried the CC genotype, while 155 had the CT/TT genotype. The CT/TT genotype was associated with a higher slope of CD4^+^ T-cells recovery (arithmetic mean ratio; AMR = 1.16; *p* = 0.016), higher CD4^+^ T-cells increase (AMR = 1.19; *p* = 0.004), and higher CD4^+^ T-cells count at the end of follow-up (AMR = 1.13; *p* = 0.006). Besides, rs6897932 CT/TT was related to a higher odds of having a value of CD4^+^ T-cells at the end of follow-up ≥500 CD4^+^ cells/mm^3^ (OR = 2.44; *p* = 0.006). After multiple testing correction (Benjamini–Hochberg), only the increase of ≥ 400 CD4^+^ cells/mm^3^ lost statistical significance (*p* = 0.052). *IL7RA* rs6897932 CT/TT genotype was related to a better CD4^+^ T-cells recovery and it could be used to improve the management of HIV-infected patients starting cART with CD4^+^ T-cells count <200 cells/mm^3^.

## 1. Background

The majority of HIV-infected individuals on combination antiretroviral therapy (cART) achieve undetectable levels of plasma HIV-RNA, recover CD4^+^ T-cell levels in peripheral blood, and restore many immunological functions [1,2]. However, a significant number of cART-treated patients fail to achieve substantial increases in the CD4^+^ T-cell count, remaining at risk of progression to acquired immune deficiency syndrome (AIDS), non-AIDS morbidities, and death [3,4,5]. 

The underlying mechanisms to this phenomenon are complex and likely multifactorial, including clinical features, such as age [6], hepatitis coinfection [7], severe immunodeficiency at the time of cART initiation [8], or a low CD4^+^ T-cells nadir [9]. Among the immunological factors associated with a poor restoration of CD4^+^ T-cells, chronic immune activation [10], levels of T-cell apoptosis [8], and reduced thymic output [11] have already been described. 

Furthermore, genetic factors have been related to CD4 T-cell restoration after virological suppression, among them specific mitochondrial haplogroups [12] and polymorphisms in genes encoding cytokines or cytokine receptors [13,14,15]. Taken together, all these factors could potentially be employed to predict impaired CD4 T-cell recovery and avoid clinical complications. Interleukin-7 (IL-7) and IL-7 receptor (IL-7R) are essential factors for T cell homeostasis by promoting development, survival, proliferation, and de novo production of T and B lymphocytes [16]. Besides, IL-7 has been described as a critical factor for CD4^+^ T-cells recovery in HIV-infected patients on cART [17]. IL-7R is a heterodimer formed by the common cytokine receptor γ-chain (CD132) and the α-chain specific of the IL-7 receptor (IL7Rα or CD127) [16]. Polymorphisms in *IL7RA*, the gene encoding for IL7Rα, have previously been associated with rapid progression to AIDS [18] and with CD4^+^ T-cell recovery after initiation of cART, especially the missense polymorphism rs6897932 [19,20,21,22]. The *IL7RA* rs6897932 Single Nucleotide Polymorphism (SNP) is a missense variant located at the *IL7RA* gene, within the alternatively spliced exon 6. This change (C/T) causes a substitution of threonine with isoleucine (Ile244) in the transmembrane region of the protein [23]. The C allele is related to alternative splicing of IL7RA that promotes an increase of the ratio between soluble IL7RA (sIL-7Rα) and membrane-bound IL-7RA (mIL-7Rα) [21,23,24]. However, no studies so far have analyzed the potential impact of this polymorphism on CD4^+^ T-cell restoration in a particular population of patients starting cART with very low CD4^+^ T-cell counts, a population of patients that is growing due to late HIV diagnosis [9] and in whom the prevalence of impaired CD4 restoration is very high [8]. 

## 2. Objective

We aimed to analyze the association between *IL7RA* rs6897932 SNP and CD4^+^ T-cell count gain in naïve patients infected with HIV who started cART with very low CD4^+^ T-cell counts (<200 cells/mm^3^). 

## 3. Methods

### 3.1. Study Population

**Figure 1:**
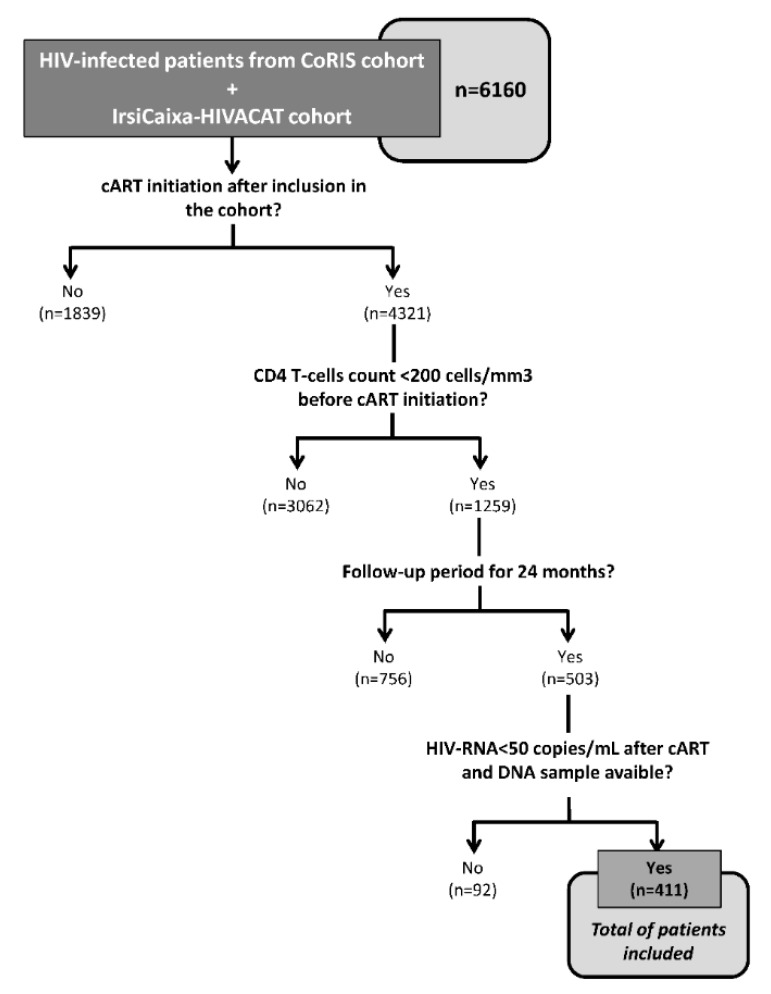
Diagram of inclusion criteria of patients. This picture shows the inclusion criteria and the sequential strategy of selection of patients included in the study. “*n*” indicates the number of patients selected after each step during filtering process.

We performed a retrospective study in 411 HIV-infected patients starting cART included in two different cohorts: The majority of patients came from the Spanish AIDS Research Network cohort (CoRIS); the rest of the patients came from the AIDS Research Institute IrsiCaixa-HIVACAT, Institut de Recerca en Ciències de la Salut Germans Trias i Pujol (Barcelona, Spain) cohort. All subjects provided informed consent to participate in the study, and the study protocol was approved by the Fundación Jiménez Díaz Ethics Committee in concordance with the Declaration of Helsinki (approval date: 26 May 2015; record number: PIC 52/2015_FJD). The inclusion criteria were: (i) Naïve for cART at inclusion in the cohort; (ii) plasma HIV-RNA > 200 copies/mL; (iii) starting cART with CD4^+^ T-cells count < 200 cells/mm^3^; (iv) complete viral suppression (plasma HIV-RNA < 50 copies/mL) for two years after starting cART; (v) regular follow up of CD4^+^ T-cells count and plasma HIV-RNA for two years after starting cART; and (vi) DNA sample available for genotyping (Figure 1). From a total of 6160 HIV-infected patients included in the CoRIS and HIVACAT cohorts, 411 patients meeting all the inclusion criteria were analyzed.

Clinical data from each patient were collected from medical records, which included demographic, clinical, virological, and laboratory data. Gender was assessed by self-identification and there were no transgender individuals. Time since HIV diagnosis was the time since the first blood test positive for HIV. The mode of transmission was recovered from the medical record of the patients and was inferred from the history of intravenous drugs use and of sexual behavior. Hepatitis B and Hepatitis C coinfection were determined by a clinical test. The clinical management of patients during follow-up was performed according to clinical guidelines.

### 3.2. DNA Genotyping

Samples were processed and frozen immediately after their reception in the HIV HGM BioBank (http://hivhgmbiobank.com/?lang=en). Total DNA isolation was performed from peripheral blood mononuclear cells with Qiagen columns (QIAamp DNA Blood Midi/Maxi; Qiagen, Hilden, Germany). DNA samples were genotyped at the Spanish National Genotyping Center (CeGen; http://www.cegen.org/) using the iPLEX^®^ Gold technology and Sequenom’s MassARRAY platform (San Diego, CA, USA).

### 3.3. Outcome Variables

The study period was 24 months. The primary outcome was the change in values of CD4^+^ T-cells during the follow-up. The outcome variables analyzed were of two types: a) Continuous: Slope of CD4^+^ T-cells and change in CD4^+^ T-cells (ΔCD4^+^) during follow-up; and CD4^+^ T-cells count at the end of follow-up; b) dichotomous: slope of CD4^+^ T-cells ≥10 and ≥15 CD4^+^ T-cells/mm^3^/month; increases of ≥200, ≥300, ≥400, and ≥500 CD4^+^ T-cells/mm^3^; and CD4^+^ T-cells at the end of follow-up ≥350 and ≥500 CD4^+^ T-cells/mm^3^.

### 3.4. Statistical Analysis

Statistical analysis was performed with Statistical Package for the Social Sciences (SPSS) software (version 22.0, SPSS INC, Chicago, IL, USA). All tests were two-tailed with *p*-values < 0.05 considered significant. Categorical data and proportions were analyzed by using a Chi-squared test or Fisher´s exact test. Mann–Whitney U test was used to compare data between independent groups when the variables were continuous. *IL7RA* rs6897932 SNP was evaluated for Hardy–Weinberg equilibrium (HWE) by Chi-square test, considering equilibrium when *p* > 0.05. We used the generalized linear model (GLM) to analyze the genetic association between *IL7RA* rs6897932 SNP and outcome variables. Specifically, a GLM with a gamma distribution (log-link) was used for continuous variables, and a GLM with a binomial distribution (logit-link) was used for dichotomous variables. These tests provide the differences between groups, the arithmetic mean ratio (AMR), and the odds ratio (OR). The multivariate regression tests were adjusted by the main clinical characteristics at baseline: (i) Dichotomous variables: Gender, HIV transmission by intravenous drugs use (IDU), hepatitis C and hepatitis B coinfection, cART regimen with protease inhibitors (PI), and Caucasian origin; (ii) continuous variables: Age, time since HIV diagnosis, and baseline CD4^+^ T-cells/mm^3^. Additionally, *p*-values were corrected for multiple testing using the false discovery rate (FDR) with Benjamini and Hochberg (*q*-values) procedure to reduce the risk of spurious results.

## 4. Results

### 4.1. Characteristics of the Study Population

The baseline characteristics of patients stratified by *IL7RA* rs6897932 genotypes are shown in Table 1. Of the total group of patients meeting the inclusion criteria, 256 subjects carried the CC genotype, while 155 carried the CT/TT genotype. Patients carrying the CT/TT genotype had a higher percentage of male (*p* = 0.023) and of subjects of Caucasian origin (*p* = 0.002) but these variables were included in the multivariate models. No significant differences were observed between both groups of patients for the rest of the epidemiological and clinical features analyzed.

### 4.2. IL7RA rs6897932 Polymorphism and CD4^+^ T-Cells Recovery

The rs6897932 SNP displayed <5% of missing values and was in Hardy–Weinberg equilibrium (*p* = 0.550). The frequency of the C allele was 79.4% and that of the T allele was 20.6%, which were in accordance with the NCBI SNP database (https://www.ncbi.nlm.nih.gov/snp/rs6897932). Thus, in the general European population, the rs6897932 T allelic frequency was 27.1% in the 1000 Genomes Project phase3 (1000Genomes) and 28.2% in the genome Aggregation Database (gnomAD-Genomes).

Table 2 shows the association between rs6897932 SNP and CD4^+^ T-cell recovery (full data of GLM models in the Supplementary file). Overall, the increases in CD4^+^ T-cells were higher in patients carrying rs6897932 CT/TT genotype (CT/TT versus CC). Thereby, in the adjusted analysis, patients carrying rs6897932 CT/TT genotype had a higher slope of CD4^+^ T-cells (AMR = 1.16; *p* = 0.016), a higher CD4^+^ T-cells increase (AMR = 1.19; *p* = 0.004), and a higher CD4^+^ T-cells count at the end of follow-up (AMR = 1.13; *p* = 0.006). Besides, patients carrying rs6897932 CT/TT had a higher odds of having a slope of CD4^+^ T-cells ≥10 CD4^+^ T-cells/mm^3^/month (OR = 1.75; *p* = 0.010) and ≥15 CD4^+^ T-cells/mm^3^/month (OR = 1.94; *p* = 0.015); a CD4^+^ T-cells increase ≥200 CD4^+^ T-cells/mm^3^ (OR = 1.63; *p* = 0.036), ≥300 CD4^+^ T-cells/mm^3^ (OR = 1.63; *p* = 0.025), ≥400 CD4^+^ T-cells/mm^3^ (OR = 1.63; *p* = 0.047), and ≥500 CD4^+^ T-cells/mm^3^ (OR = 2.16; *p* = 0.018); and a value of CD4^+^ T-cells at the end of follow-up ≥500 CD4^+^ T-cells/mm^3^ (OR = 2.44; *p* = 0.006). These *p*-values were corrected for multiple testing using the false discovery rate (FDR) with the Benjamini and Hochberg procedure, and after doing this correction, the statistical significance was lost only for the outcome variable of CD4^+^ T-cells increase ≥400 CD4^+^ cells/mm^3^ (*q*-value = 0.052).

## 5. Discussion

In this study, HIV-infected patients carrying *IL7RA* rs6897932 CT/TT genotype had a better CD4^+^ T-cell count recovery after starting cART with <200 CD4 T-cells/mm^3^. This association was found for almost all the outcome variables analyzed, both in the univariate and in the multivariate analysis adjusted by the main baseline characteristics. It should be noted that a large number of outcome variables, both continuous and dichotomous, were analyzed, which reflect different effects of immune reconstitution.

Our study supports the positive impact of rs6897932 T allele on CD4^+^ T-cell count recovery in cART-treated patients, in agreement with previous studies performed in cohorts of patients with different ethnicity and different inclusion criteria [19,20,22]. The study of Guzmán-Fulgencio et al. [19] was performed in the Spanish population, but we want to emphasize some significant differences concerning our study. In the previous report of Guzmán-Fulgencio et al. [19], they studied a cohort of HIV-infected patients who had baseline CD4^+^ T-cells values <350 cells/mm^3^, from a single Spanish reference hospital, and with a non-uniform follow-up. In the current study, we analyzed a sample more representative of the Spanish population (our cohort of patients infected with HIV came from a large number of hospitals spread throughout Spain), we applied a more restricted inclusion criteria (all patients included had a baseline CD4^+^ T-cell count <200 cells/mm^3^ and undetectable plasma HIV-RNA during the whole follow-up period), and the follow-up period was the same in all patients (24 months after starting cART). In addition, the statistical analysis applied was different in both studies. In the report of Guzmán-Fulgencio et al. [19], a survival analysis was performed with CD4^+^ T-cell count >500 cells/mm^3^ as the primary outcome, whereas in the present study, we evaluated the changes in the CD4^+^ T-cell count during a period of 24 months and used different threshold values to evaluate the degree of CD4^+^ T-cells reconstitution.

The *IL7RA* rs6897932 C allele has been associated to an increase of the ratio between sIL-7Rα and mIL-7Rα [21,23,24], leading to a reduction of the bioavailability of IL-7 and limiting its effects [25]. By contrast, rs6897932 T allele has been associated with lower plasma levels of sIL-7Rα [21,23,24], without limiting the effect of circulating IL-7 [25]. Besides, in an in vitro model, Lundtoft et al. identified a dominant effect of the protective *IL7RA* haplotype tagged by rs6897932 on mIL-7Rα expression, whereas the risk *IL7RA* haplotype mainly affected the sIL-7Rα [26]. In HIV-infected patients, rs6897932 CC genotype is associated with higher plasma levels of sIL7RA [21,24], whereas rs6897932 TT genotype is related to lower plasma levels of sIL7RA [21,24]. Since the CD4^+^ T-cell count recovery is predominantly driven by increases in CD4^+^CD127^+^ T cells in HIV-infected patients on cART [27], rs6897932 T carriers may have an advantage because the IL-7 bioavailability is increased. Moreover, in a recent article, Hartling et al. [28] also suggested that the effect of rs6897932 is driven by an increased response of IL-7R to IL-7 in patients with TT genotype, which is in line with a faster CD4^+^ T-cell recovery in carriers of the T allele [19,20,21]. Thus, the TT genotype was associated with increased signal transduction and proliferation in response to IL-7 among HIV-infected individuals [28]. As discussed above, the rs6897932 T allele, versus C allele, may confer lower sIL-7Rα levels, higher IL-7 bioavailability, and higher capacity of CD4^+^ T-cell recovery, leading to a greater immune response against HIV infection. However, in our study, we did not have any direct functional measurements of *IL7RA* rs6897932 SNP to provide additional data on the potential mechanism.

Our data suggest that *IL7RA* rs6897932 SNP may have an impact on clinical practice in the population of HIV-infected patients beginning cART with very low CD4^+^ T-cell count, a population that is growing due to delayed HIV diagnosis [9,29] and in whom impaired CD4 restoration is widespread [8,30]. Thus, the CT/TT genotype is related to the immunological responders (IRs) patients that may recover CD4^+^ T-cell counts to optimal levels 24 months after starting cART, whereas the CC genotype is related to the immunological non-responders (INRs) patients that present a low increase in CD4 T-cell counts despite successful cART and viral suppression. *IL7RA* rs6897932 SNP may help to identify those patients who are at a higher risk of being INR in whom implementation of adjuvant therapies may be needed to improve immune reconstitution and to prevent disease progression and death.

## 6. Study Limitations

Various limitations of our study need to be taken into account. Firstly, the retrospective design may impose a selection bias and prevent the inclusion of other potential confounding variables. Secondly, the limited sample size may have impaired the ability to detect less robust associations. Thirdly, this study was mostly carried out on Caucasian subjects and our conclusions are only truly applicable to this population.

## 7. Conclusions

In summary, the *IL7RA* rs6897932 CT/TT genotype was related to a better CD4^+^ T-cell count recovery after 24 months of therapy in HIV-infected patients who started cART with a CD4^+^ T-cell count <200 cells/mm^3^. Therefore, our findings could provide information to improve the management of HIV-infected patients with poor prognosis of CD4^+^ T-cell recovery.

## Figures and Tables

**Table 1:** Clinical and epidemiological characteristics at baseline of HIV infected patients who started combination antiretroviral therapy with very low CD4^+^ T-cells count (<200 cells/mm^3^).

Characteristics	All Patients	*IL7RA* rs6897932 Genotypes		
		CC	CT/TT	*p*-Value
No.	411	256	155	
Male (n = 411) (%)	323 (78.6%)	192 (75%)	131 (84.5%)	**0.023**
Age (n = 411) (years)	40 (34; 48)	40 (34; 46)	50 (33; 49)	0.488
Caucasian origin (n = 394) (%)	317 (80.5%)	187 (75.7%)	130 (88.4%)	**0.002**
Time since HIV diagnosis (n = 411) (years)	1 (1; 1)	1 (1; 1)	1 (1; 1)	0.517
CD4^+^ cell count at baseline (n = 411) (cells/μL)	104 (41; 159)	92.7 (38; 157)	115 (47; 162)	0.198
Hepatitis C infection (n = 411) (%)	32 (7.8%)	23 (9%)	9 (5.8%)	0.244
Hepatitis B infection (n = 411) (%)	20 (4.9%)	14 (5.5%)	6 (3.9%)	0.466
cART regimen (n = 411) (%)				0.054
PI-based	127 (31%)	73 (28.5%)	54 (35.1%)	
NNRTI-based	205 (50%)	134 (52.3%)	71 (46.1%)	
PI+NNRTI-based	53 (12.9%)	38 (14.8%)	15 (9.7%)	
Others	25 (6.1%)	11 (4.4%)	14 (9.1%)	
HIV transmission route (n = 384) (%)				0.079
Homosexual transmission	189 (49.2%)	106 (45.1%)	83 (55.7%)	
Heterosexual transmission	139 (36.2%)	95 (40.4%)	44 (29.5%)	
IDU	56 (14.6%)	34 (14.5%)	22 (14.8%)	

Statistical: Values were expressed as absolute number (percentage) and median (percentile 25; percentile 75). Significant differences are shown in bold. The percentages were calculated with respect to the available values, which are indicated in the left column in parentheses. *p*-values were calculated by Chi-square and Mann–Whitney tests. Abbreviations: IDU, intravenous drug users; HIV, Human immunodeficiency virus; cART, combination antiretroviral therapy; PI, HIV protease inhibitor; NNRTI, non-nucleoside analogue HIV reverse transcriptase inhibitor.

**Table 2:** Summary of the CD4^+^ T-cells recovery according to *IL7RA* rs6897932 polymorphism in HIV-infected patients who started combination antiretroviral therapy with very low CD4^+^ T-cells count (<200 cells/mm^3^).

Outcomes	rs6897932 Genotypes ^(^*^)^		Unadjusted Analysis ^(^*^)^			Adjusted Analysis ^(^**^)^		
	CC (*n* = 256)	CT/TT (*n* = 155)	Exp(B) (95% CI)	*p*-Value	*q*-Value	Exp(B) (95% CI)	*p*-Value	*q*-Value
Slope CD4^+^ recovery	9.1 (5.9; 12.9)	10.3 (6.1; 14.8)	1.12 (0.99; 1.28)	0.070	0.070	1.16 (1.03; 1.31)	**0.016**	0.028
≥10 CD4^+^ cells/mm^3^ per month	105 (41%)	82 (52.9%)	1.61 (1.08; 2.41)	**0.019**	**0.044**	1.75 (1.14; 2.69)	**0.010**	**0.028**
≥15 CD4^+^ cells/mm^3^ per month	38 (14.8%)	37 (23.9%)	1.79 (1.08; 2.88)	**0.023**	**0.044**	1.94 (1.14; 3.30)	**0.015**	**0.028**
CD4^+^ increase (ΔCD4^+^)	258 (167; 381)	295 (189; 442)	1.15 (1.02; 1.31)	**0.020**	**0.044**	1.19 (1.06; 1.34)	**0.004**	**0.022**
≥200 CD4^+^ cells/mm^3^	164 (64.1%)	114 (73.5%)	1.56 (1.01; 2.42)	**0.047**	0.054	1.63 (1.03; 2.57)	**0.036**	**0.044**
≥300 CD4^+^ cells/mm^3^	97 (37.9%)	75 (44.8%)	1.53 (1.03; 2.30)	**0.037**	0.051	1.63 (1.06; 2.49)	**0.025**	**0.034**
≥400 CD4^+^ cells/mm^3^	54 (21.1%)	48 (31%)	1.67 (1.06; 2.64)	**0.025**	**0.044**	1.63 (1.01; 2.63)	**0.047**	0.052
≥500 CD4^+^ cells/mm^3^	24 (9.4%)	26 (16.8%)	1.94 (1.06; 3.53)	**0.028**	**0.044**	2.16 (1.14; 4.11)	**0.018**	**0.028**
CD4^+^ at the end of follow-up	362 (260; 463)	425 (274; 558)	1.14 (1.03; 1.25)	**0.009**	**0.044**	1.13 (1.03; 1.24)	**0.006**	**0.022**
≥350 CD4^+^ cells/mm^3^	133 (52%)	96 (61%)	1.51 (1.01; 2.26)	**0.049**	0.054	1.51 (0.96; 2.37)	0.077	0.077
≥500 CD4^+^ cells/mm^3^	52 (20.3%)	59 (38.1%)	2.41 (1.54; 3.76)	**<0.001**	**0.001**	2.44 (1.49; 3.99)	**0.006**	**0.022**

Statistical: (*) Values were expressed as absolute number (percentage) and median (percentile 25; percentile 75). (**), *p*-values were calculated by univariate regression or multivariate regression adjusted by the most important clinical and epidemiological characteristics (see statistical analysis section). *p*-values, raw *p*-values; *q*-values, *p*-values corrected for multiple testing using the false discovery rate (FDR) with the Benjamini and Hochberg procedure. The statistically significant differences are shown in bold. Significant differences are shown in bold. Abbreviations: Exp(B), exponentiation of the B coefficient, which was an arithmetic mean ratio (AMR) for continuous variable and an odds ratio (OR) for categorical variables; 95%CI, 95% of confidence interval; *p*-value, level of significance; HIV, human immunodeficiency virus; aAMR, adjusted arithmetic mean ratio; IL7RA, interleukin 7 receptor α-chain.

## Data Availability

The datasets used and analyzed during the current study may be made available by the corresponding author, upon reasoned request.

## Supplementary Materials

The following are available online at https://www.mdpi.com/2218-273X/9/6/233/s1.

## Appendix A

*“Centers and Investigators Involved in CoRIS”*

**Executive committee**: Santiago Moreno, Inma Jarrín, David Dalmau, Maria Luisa Navarro, Maria Isabel González, Jose Luis Blanco, Federico Garcia, Rafael Rubio, Jose Antonio Iribarren, Félix Gutiérrez, Francesc Vidal, Juan Berenguer, Juan González.

**Fieldwork, data management and analysis**: Inma Jarrín, Belén Alejos, Victoria Hernando, Cristina Moreno, Carlos Iniesta, Luis Miguel Garcia Sousa, Nieves Sanz Perez.

**HIV BioBanK:** Hospital General Universitario Gregorio Marañón: M Ángeles Muñoz-Fernández, Isabel María García-Merino, Irene Consuegra Fernández, Coral Gómez Rico, Jorge Gallego de la Fuente, Paula Palau Concejo.

*Participating centres:*

**Hospital General Universitario de Alicante (Alicante):** Joaquín Portilla, Esperanza Merino, Sergio Reus, Vicente Boix, Livia Giner, Carmen Gadea, Irene Portilla, María Pampliega, Marcos Díez, Juan Carlos Rodríguez, José Sánchez-Payá.

**Hospital Universitario de Canarias (San Cristobal de la Laguna):** Juan Luis Gómez, Jehovana Hernández, María Remedios Alemán, María del Mar Alonso, María Inmaculada Hernández, Felicitas Díaz-Flores, Dácil García, Ricardo Pelazas, Ana López Lirola.

**Hospital Universitario Central de Asturias (Oviedo):** José Sanz Moreno, Alberto Arranz Caso, Cristina Hernández Gutiérrez, María Novella Mena.

**Hospital Universitario 12 de Octubre (Madrid):** Rafael Rubio, Federico Pulido, Otilia Bisbal, Asunción Hernando, Lourdes Domínguez, David Rial Crestelo, Laura Bermejo, Mireia Santacreu.

**Hospital Universitario de Donostia (Donostia-San Sebastián):** José Antonio Iribarren, Julio Arrizabalaga, María José Aramburu, Xabier Camino, Francisco Rodríguez-Arrondo, Miguel Ángel von Wichmann, Lidia Pascual Tomé, Miguel Ángel Goenaga, Mª Jesús Bustinduy, Harkaitz Azkune, Maialen Ibarguren, Aitziber Lizardi, Xabier Kortajarena.

**Hospital General Universitario De Elche (Elche):** Félix Gutiérrez, Mar Masiá, Sergio Padilla, Andrés Navarro, Fernando Montolio, Catalina Robledano, Joan Gregori Colomé, Araceli Adsuar, Rafael Pascual, Marta Fernández, Elena García, José Alberto García, Xavier Barber.

**Hospital General Universitario Gregorio Marañón (Madrid):** Juan Berenguer, Juan Carlos López Bernaldo de Quirós, Isabel Gutiérrez, Margarita Ramírez, Belén Padilla, Paloma Gijón, Teresa Aldamiz-Echevarría, Francisco Tejerina, Francisco José Parras, Pascual Balsalobre, Cristina Diez, Leire Pérez Latorre.

**Hospital Universitari de Tarragona Joan XXIII (Tarragona):** Francesc Vidal, Joaquín Peraire, Consuelo Viladés, Sergio Veloso, Montserrat Vargas, Miguel López-Dupla, Montserrat Olona, Anna Rull, Esther Rodríguez-Gallego, Verónica Alba.

**Hospital Universitario y Politécnico de La Fe (Valencia):** Marta Montero Alonso, José López Aldeguer, Marino Blanes Juliá, María Tasias Pitarch, Iván Castro Hernández, Eva Calabuig Muñoz, Sandra Cuéllar Tovar, Miguel Salavert Lletí, Juan Fernández Navarro.

**Hospital Universitario La Paz/IdiPAZ:** Juan González-garcia, Francisco Arnalich, José Ramón Arribas, Jose Ignacio Bernardino de la Serna, Juan Miguel Castro, Luis Escosa, Pedro Herranz, Victor Hontañón, Silvia García-Bujalance, Milagros García López-Hortelano, Alicia González-Baeza, Maria Luz Martín-Carbonero, Mario Mayoral, Maria Jose Mellado, Rafael Esteban Micán, Rocio Montejano, María Luisa Montes, Victoria Moreno, Ignacio Pérez-Valero, Berta Rodés, Talia Sainz, Elena Sendagorta, Natalia Stella Alcáriz, Eulalia Valencia.

**Hospital San Pedro Centro de Investigación Biomédica de La Rioja (CIBIR) (Logroño):** José Ramón Blanco, José Antonio Oteo, Valvanera Ibarra, Luis Metola, Mercedes Sanz, Laura Pérez-Martínez.

**Hospital Universitari MutuaTerrassa (Terrasa):** David Dalmau, Angels Jaén, Montse Sanmartí, Mireia Cairó, Javier Martinez-Lacasa, Pablo Velli, Roser Font, Mariona Xercavins, Noemí Alonso.

**Complejo Hospitalario de Navarra (Pamplona)** María Rivero, Jesús Repáraz, María Gracia Ruiz de Alda, María Teresa de León Cano, Beatriz Pierola Ruiz de Galarreta.

**Corporació Sanitària Parc Taulí (Sabadell):** Ferrán Segura, María José Amengual, Gemma Navarro, Montserrat Sala, Manuel Cervantes, Valentín Pineda, Sonia Calzado, Marta Navarro.

**Hospital Universitario de La Princesa (Madrid):** Ignacio de los Santos, Jesús Sanz Sanz, Ana Salas Aparicio, Cristina Sarriá Cepeda, Lucio Garcia-Fraile Fraile, Enrique Martín Gayo.

**Hospital Universitario Ramón y Cajal (Madrid):** Santiago Moreno, José Luis Casado, Fernando Dronda, Ana Moreno, María Jesús Pérez Elías, Cristina Gómez Ayerbe, Carolina Gutiérrez, Nadia Madrid, Santos del Campo Terrón, Paloma Martí, Uxua Ansa, Sergio Serrano, María Jesús Vivancos.

**Hospital General Universitario Reina Sofía (Murcia)** Enrique Bernal, Alfredo Cano, Antonia Alcaraz García, Joaquín Bravo Urbieta, Ángeles Muñoz, Maria Jose Alcaraz, Maria del Carmen Villalba.

**Hospital Nuevo San Cecilio (Granada):** Federico García, José Hernández, Alejandro Peña, Leopoldo Muñoz, Paz Casas, Marta Alvarez, Natalia Chueca, David Vinuesa, Clara Martinez-Montes.

**Centro Sanitario Sandoval (Madrid):** Jorge Del Romero, Carmen Rodríguez, Teresa Puerta, Juan Carlos Carrió, Mar Vera, Juan Ballesteros, Oskar Ayerdi.

**Hospital Universitario Son Espases (Palma de Mallorca):** Melchor Riera, María Peñaranda, María Leyes, Mª Angels Ribas, Antoni A Campins, Carmen Vidal, Francisco Fanjul, Javier Murillas, Francisco Homar.

**Hospital Universitario Virgen de la Victoria (Málaga):** Jesús Santos, Crisitina Gómez Ayerbe, Isabel Viciana, Rosario Palacios, Carmen María González.

**Hospital Universitario Virgen del Rocío (Sevilla):** Pompeyo Viciana, Nuria Espinosa, Luis Fernando López-Cortés.

**Hospital Universitario de Bellvitge (Hospitalet de Llobregat):** Daniel Podzamczer, Elena Ferrer, Arkaitz Imaz, Juan Tiraboschi, Ana Silva, María Saumoy.

**Hospital Costa del Sol (Marbella):** Julián Olalla, Alfonso del Arco, Javier de la torre, José Luis Prada, José María García de Lomas Guerrero, Javier Pérez Stachowski.

**Hospital General Universitario Santa Lucía (Cartagena):** Onofre Juan Martínez, Francisco Jesús Vera, Lorena Martínez, Josefina García, Begoña Alcaraz, Amaya Jimeno.

Complejo Hospitalario Universitario a Coruña (Chuac) (A Coruña): Angeles Castro Iglesias, Berta Pernas Souto, Alvaro Mena de Cea.

**Hospital Universitario Virgen de la Arrixaca (El Palmar):** Carlos Galera, Helena Albendin, Aurora Pérez, Asunción Iborra, Antonio Moreno, Maria Angustias Merlos, Asunción Vidal.

**Hospital Universitario Infanta Sofia (San Sebastian de los Reyes):** Inés Suárez-García, Eduardo Malmierca, Patricia González-Ruano, Dolores Martín Rodrigo, Mª Pilar Ruiz Seco.

**Complejo Hospitalario de Jaén (Jaén)** Mohamed Omar Mohamed-Balghata, María Amparo Gómez Vidal.

**Hospital Clínico San Carlos (Madrid):** Vicente Estrada Pérez, Maria Jesus Téllez Molina, Jorge Vergas García, Juncal Pérez-Somarriba Moreno.

**Hospital Universitario Fundación Jiménez Díaz (Madrid):** Miguel Górgolas., Alfonso Cabello., Beatriz Álvarez., Laura Prieto.

**Hospital Universitario Príncipe de Asturias (Alcalá de Henares):** José Sanz Moreno, Alberto Arranz Caso, Cristina Hernández Gutiérrez, María Novella Mena.

**Hospital Clínico Universitario de Valencia (València):** María José Galindo Puerto, Ramón Fernando Vilalta, Ana Ferrer Ribera.

**Hospital Reina Sofía (Córdoba):** Antonio Rivero Román, Maria Teresa Brieva Herrero, Antonio Rivero Juárez, Pedro López López, Isabel Machuca Sánchez, José Peña Martínez.

**Hospital Universitario Severo Ochoa (Leganés)**: Miguel Cervero Jiménez, Rafael Torres Perea, Juan José Jusdado Ruiz-Capillas.

Nuestra Señora de Valme (Sevilla): Juan A Pineda.
